# The Hospital. Nursing Section

**Published:** 1906-11-10

**Authors:** 


### The Hospital.

**Nursing Section.**

Contributions for "THE HOSPITAL," should be addressed to the EDITOR, "THE HOSPITAL" NURSING SECTION, 28 & 29 Southampton Street, Strand, London, W.C.

No. 1,051.—VOL. XLI. SATURDAY, NOVEMBER 10, 1906.

### Notes on News from the Nursing World.

### THE NATURAL INSTINCTS OF THE SECRETARY FOR WAR.

THE Secretary of State for War supplied the House of Commons on Thursday evening last week with the result of his personal investigations into the amusements of Army nurses. He stated that he had consulted the Nursing Board, which, he said, contained amongst its members many ladies of great experience in the arrangements which had been made for the performance of the duties of the nurses. The information he received from the Board was that the nursing service requires gentlewomen who are devoted first and foremost to their work for its own sake and the sake of their patients; and that while occasional attendance at operas, theatres, concerts, and other places of amusement is not considered incompatible with the performance of their duties and is allowed at the discretion of the matron, the late hours involved in attendance at balls and dances incapacitates them from giving proper attention to their patients on the following day. Mr. Haldane added the confession that the weight and authority of "experienced matrons and highly experienced ladies had prevailed over his natural instincts." But if Army nurses themselves do not desire the removal of the rule prohibiting them from attending public balls, there is no reason why the natural instincts of the right hon. gentleman should be allowed to prevail. An alternative is the substitution of a rule simply requiring nurses on leave to return to their quarters by 12 A.M.

### HAIR PADS AND JEWELLERY.

THE Inspector-General of Hospitals in New Zealand refers in his annual report to Parliament to the subject of nurses' uniforms. Observing that washing dresses, white aprons, and removable sleeves represent the absolute cleanliness and neatness, together with the freedom of movement required in carrying out the duties of a sick nurse, he says that of late he has noticed with regret an increasing tendency for nurses on duty to wear dangling chains, numerous brooches, and rings, and that even some hospital matrons adopt this habit. The Inspector-General himself counted as many as five brooches worn at the same time by a matron in the North Island. The nurses' cap, he affirms, has shrunk from a covering for the hair into a small piece of starched linen, crowning an edifice of pads and loose hair. He condemns the matron for permitting this, and accuses of inconsistency the surgeon who is particular about the scrubbing of hands and arms, but submits to untidy heads of hair in the ward and in the operating-room. To emphasise the prevalence of the practices which he condemns, the Inspector-General exempts the Wellington Hospital from his strictures, and says that the nurses being trained in that institution are neat and tidy in uniform, and are not allowed to wear hair-pads and jewellery whilst on duty. It is fair to add that the authorities of the Auckland Hospital, traversing the allegations of the Inspector-General, affirm that very few hair-pads are worn and practically no jewellery. A nurse in the latter hospital who was charged with wearing an enormous pad held her peace until the accuser had finished, and then, removing her hair-pins let down her hair, and—it was all her own.

### THE TITLE OF LADY.

WE cannot allow the letter from "A Sister," which appears in another column under the title of "The Standard of Nursing," to pass without remark. In urging that nurses should be "treated as ladies," and "not as a porter's wife could expect to be treated," our correspondent reflects the kind of feeling which, we are afraid, is far too prevalent. If a porter's wife were rudely treated the discredit would attach to others, not to herself. She has as much claim to courtesy as a nurse of the highest birth, and there is no reason why her behaviour and qualifications should not be all that could be desired. If they were not, she could be dismissed.

### THE HOSTEL CONTROVERSY.

AS we intimate in another column, we cannot publish further correspondence respecting the Nurses' Hostel, but if any new issue of importance arises we shall, of course, be prepared to deal with it on its merits. It will have been observed that we have allowed both sides to have a fair hearing; and so far as the management is concerned, we freely recognise that there have been things to praise as well as to blame. Fault, we dare say, has been unreasonably found in some cases, but it seems clearly established that, in the past, at any rate, the idea of maintaining discipline has been pushed to the extreme when the main object in view should be to render the guests at the Hostel comfortable. A letter which we publish to-day tends to justify the conclusion that it is intended to evict by degrees from the Hostel the nurses who signed the petition which was so distasteful to the Board. We remain silent with respect to the original cause of all the controversy—the apparently inexplicable dismissal of Miss Hulme from the office of lady superintendent—because that question may again come before our readers later on.

### IRRELEVANT QUESTIONS.

The Guardians of Richmond, Surrey, have just appointed a nurse. But prior to the selection several applicants for the post were asked to appear before the Board, and, of course, a number of questions were put to them. But we think that great care should be taken to avoid interrogations of a certain character. Thus, at Richmond, applicants were requested to state whether they were total abstainers, whether they were engaged to be married, and what their religious views were. These are questions which savour of impertinence, and are distinctly irrelevant. They also suggest an undue interference to which nurses have a perfect right to object.

### NURSING ASSOCIATIONS AND MIDWIFERY CASES.

At the annual meeting of the Cumberland Nursing Association the other day Lady Mabel Howard moved that, in complicated midwifery cases, when nurses called in the assistance of doctors, the Association should guarantee the expenses of the medical men at the rate of 10s. a visit. The Dean of Carlisle, in seconding the motion, explained that he only did so in order to provoke discussion, and both Canon Rawnsley and Dr. Graham opposed it—the Canon on the ground that it was a most dangerous principle to establish, and Dr. Graham because in such cases as those indicated by Lady Mabel Howard application could be made to the parish doctor, and, upon a recommendation for feeing obtained from the relieving officer, the medical man was paid a fee of £2 for this attention. The rejoinder of Lady Mabel was that the parish doctor sometimes lives a considerable distance away, and she stated that she had brought forward the matter because medical men could refuse, and had refused, to render assistance without fee or for an inadequate fee. The matter was subsequently referred to the Executive Committee for consideration. Meanwhile, we may say that it would be extremely difficult to secure skilled service in these complicated cases for a fee of 10s.

### EMERGENCY NURSES AT KING'S LYNN INFIRMARY.

The King's Lynn Guardians have been compelled to engage two emergency trained nurses for the Workhouse Infirmary at a salary of £2 2s. a week each. The Local Government Board Inspector, Captain Hervey, after visiting the Infirmary at various times, entered in the visitors' book that he was not satisfied that the male patients were receiving adequate nursing attention during the night. He pointed out that as neither of the nurses on night duty had enjoyed previous experience in nursing, and the superintendent nurse was obliged to be on duty for twenty-four hours at a stretch; and he drew attention to the fact that there was a typhoid case which required the full and undivided attention of one nurse. Subsequently, at the instance of the medical officer, nurses to attend the typhoid case, one for night and one for day duty, were installed. But a permanent arrangement is clearly required. Apart from the typhoid case and the possibility of further cases, there should always be one trained nurse on duty at night; and we have no doubt that the Local Government Board will press this matter upon the Guardians until they make the needful addition to the staff. Apparently, if Captain Hervey had not intervened, the emergency nurses for the typhoid patient would not have been engaged.

### STRANGE OBJECTIONS TO TENNIS.

The Wirral Joint Hospital Board spent some time last week in discussing the question whether the nurses at Clatterbridge Hospital should be provided with a tennis-court. One of the members urged that if the request were complied with people outside would think that the nurses had very little to do; another feared that the introduction of such a game "would make things appear too happy for such an institution"; and a third demurred to the expense. The last argument was practically disposed of by the statement that there is ground available, and that the only expense would be the purchase of nets, rackets, and balls. Ultimately, the other objections were overruled, and we are glad to say that the nurses at Clatterbridge Hospital are in future to be permitted to indulge in a game of tennis when they are off duty.

### A CASE FOR BICYCLES.

It is very desirable that the nurses attached to an isolation hospital should not themselves be isolated. We regret, therefore, that the nurses at Lodge Moor Hospital, Sheffield, find it difficult, on account of their distance from town, to make good use of their off-duty time. They are two miles from a church, and such omnibus accommodation as there is does not seem to serve their purpose. We do not suppose that the omnibus proprietors will alter their arrangements to suit the convenience of a few occasional passengers. But is not this a case in which the institution might assist nurses who do not already possess bicycles to purchase them? There is no class of nurses who need a reasonable amount of recreation more than those who have to take charge of patients suffering from infectious diseases, and we hope that the authorities of Lodge Moor Hospital will be able to adopt our suggestion.

### A CONDITIONAL SUBSCRIPTION.

The Duchess of Northumberland has intimated to the Northumberland County Nursing Association that she can only continue her subscription of £100 a year on condition that the Association itself will collect in annual subscriptions a further £100. There are, it appears, fifty presidents of districts, and the Duchess suggests that each of them should obtain £2 or £3 more per year, which would give the required addition. We do not say that this is an unreasonable stipulation, though we do not altogether like the practice of making large subscriptions conditional upon the collection of small ones. It may tend to stimulate endeavour, but it does not encourage the desirable spontaneous character of the contributions.

### GUY'S HOSPITAL NURSES' PHOTOGRAPHIC SOCIETY.

On Monday last an interesting entertainment was given in the Court Room of Guy's Hospital in connection with the Nurses' Photographic Society. The Rev. Canon Horsley lectured on "The Scenery and People of the Bernese Oberland." His remarks were illustrated by exceptionally good lantern-slides, which had been prepared from photographs taken during the tours of two or three recent years. The Society is indebted to Sister "Naaman" for the arrangement of the evening and for securing the services of Canon Horsley. The lantern was managed by Miss M. Smith, Secretary of the Society. The proceeds from the sale of tickets are to be devoted to the funds of the Nurses' League.

### POOR-LAW NURSES AND SUPERANNUATION ACT.

Numerous Poor-law nurses who have contracted out under the Superannuation Act of 1896 now desire to come in on paying up the arrears of contributions from the commencement. We therefore hope that the Parliamentary Committee of the National Poor-law Officers' Association will bring their inquiry into the matter to a speedy termination. It is indisputable that though certain Boards of Guardians are allowing the course suggested to be pursued, the position requires to be more clearly defined. There ought to be no room for doubt, but so long as there is any, nurses who have contracted out of the Act had better not pay up arrears, with the risk of losing their money.

### BOLINGBROKE HOSPITAL LINEN LEAGUE.

The first annual meeting of the Bolingbroke Hospital Linen League took place last week, and was attended by a large number of ladies. The record of work was extremely encouraging. Although the League is quite in its infancy, having only been in existence for a few months, there were over 500 garments on show at the hospital as the result of the labours of the members, and over £50 had been collected in money. An interesting speech was delivered by one of the Presidents, Mrs. Makins, who spoke with authority on the subject. Before her marriage she was a nurse for some years at St. Thomas's Hospital, and she affirmed that a League which would not only supply linen for the hospital itself, but garments for the patients themselves to wear in the wards, was a badly needed institution in every hospital. It added considerably to the comfort of the sufferers, not only by keeping them warm, but by preventing worry. It much distressed the respectable poor to feel that they were more shabby or ragged than their neighbours. If all patients were clad in regulation garments provided by the hospitals this trouble, added Mrs. Makins, disappeared at once, and they were able to benefit to the full by the ministrations of doctor and nurse.

### PECKHAM NURSING ASSOCIATION.

The sixth annual meeting of this Association was held at the residence of Mr. C. Goddard Clarke, M.P. The report, which was read and adopted, showed that during the past year the Association has acted under fifty-four doctors, and dealt with fifty-four different complaints (including fifteen cases of cancer); and among the patients attended there were thirty-five from well-known London hospitals. It was also stated that the annual sale of work in June realised a net sum of £173. Mrs. Charles Ward, the foundress, referred to several severe cases of rheumatism and arthritis which were receiving attention, and said that she was very anxious to instal a well-known apparatus in the Nurses' Home for the treatment of these diseases, and hoped that funds would be forthcoming for the purpose.

### CLAPHAM SCHOOL OF MIDWIFERY EXAMINATION.

The examination under the auspices of the Clapham School of Midwifery took place at the Clapham Maternity Hospital on Saturday last. It was, as usual, written, *viva-voce*, and clinical, and the results were obtained by means of marks adjudged by the three examiners in the three separate departments each independently. Of the 22 candidates 20 satisfied the examiners, 6 passing with honours—*i.e.* with five-sixths of the maximum number of marks obtainable—and the examiners expressed the opinion that the standard this time was exceptionally high. It may be added that there was no failure at the examination of the Central Midwives Board, held a few days later, among those candidates who had passed at the Clapham examination, while the only two who failed at the one failed also at the other.

### OUR CLOTHING DISTRIBUTION.

We make no apology to our readers for reminding them that we are exceedingly anxious to receive a large number of parcels for our Clothing Distribution this year, because we are satisfied from inquiries that the need for assistance is likely to be greater than ever. Among the applications received for a participation in the distribution is one from Lancashire. The matron writes that the "smallest help" would be acceptable. In addition to the contributions already announced, we are glad to acknowledge the receipt of garments from a Convalescent Home Nurse; a Grimsby Nurse; Miss Elgar, Hunstanton; and an anonymous contributor, Horrabridge, Devonshire. All parcels must be sent addressed to the Editor, 28 and 29 Southampton Street, Strand, London, W.C., and should have "Clothing Distribution" written on the outside.

### SHORT ITEMS.

Church Army authorities are receiving the names of candidates for the next term of training for lay evangelists and mission women, beginning in January next; the training is free, board and lodging being supplied gratis, and salaried posts are guaranteed to the successful candidates.—Miss Ethel Perrin, of Stourport, successfully passed the Assistants' Examination, Apothecaries' Hall, London, held in October last; she received her preparation for it at the dispensary of the Women's Hospital and at the Technical School, Birmingham.—A meeting of the Council of Queen Victoria's Jubilee Institute for Nurses was held on Wednesday last week. Reports were received from the various committees showing steady advancement of the work of the Institute.—Miss Ada Hopwood and Miss Mary A. Leighton have passed the November examination of the Central Midwives Board. They received their practical training in the Maternity Ward of Crumpsall Infirmary, and attended the official course of lectures at St. Mary's Hospitals, Manchester.

### The Nursing Outlook.

"From magnanimity, all fears above;
From nobler recompense, above applause,
Which owes to man's short outlook all its charm."

### NURSES IN ISOLATION HOSPITALS.

The isolation or fever hospital is sometimes anything but what it should be. In a recent case, tried in Tunbridge Wells, where the friends of the patient brought an action against the local authorities, owing to the scandalous arrangements at the isolation hospital, they succeeded in getting a judgment. This fact should cause Mr. John Burns to call for a special report, after inspection, of the present condition and organisation of every small isolation hospital in the country. We are afraid this case is not by any means an isolated instance of the existence of an isolation hospital in name rather than in fact. It showed there was no proper system of management, no adequate nursing provision or attendance, and that the ward, where the patient was, was used as a wash-house and kitchen, amongst other things. The cause, no doubt, for the existence of this state of things lies in the fact, that, in smaller districts considerable periods may elapse without having cases of infectious disease to send to the so-called isolation hospitals. This circumstance does not, however, diminish in any way the responsibility which rests upon the local authority and their medical officer, to make such arrangements as will secure, that the isolation hospital, however small, shall be capable of being at once fully equipped and organised, on efficient lines, whenever it is needed for the reception of patients. Of course this means the expenditure of some money, annually, but such expenditure, under proper conditions, must prove an economy, as well as a safeguard, to the ratepayers who supply the funds. No doubt, in the greater cities and in large towns, isolation hospitals are usually suitably equipped, and reasonably well administered. In proof of this we may refer to a patient's account of his treatment at a great fever hospital in Glasgow, which appears on another page of our present issue.

We have called attention to the defects of the smaller isolation hospitals because, a recent movement, which we believe originated in the West Riding. It has now taken root in the counties of Gloucester and Worcester, including the cities of Bristol, Gloucester and Worcester. It should have a material influence for good, by making the continuous and efficient equipment of a small isolation hospital, in an outlying district, much easier and less costly, than it has ever been before. The movement, in the two latter counties, took practical shape at an influentially attended conference of members and officers of the county councils and other authorities, responsible for the local government of these counties. The conference had under consideration a nurses' exchange for isolation hospitals, in the administrative counties of Gloucester and Worcester, and the three cities just named. The practical outcome of the conference is largely due to the interest taken in the matter by Mr. W. F. Hicks Beach, Chairman of the Gloucester Sanitary Committee and by Drs. G. H. Fosbrooke and J. Middleton Martin, the county medical officers of health. At the present time it is found very difficult, and sometimes impossible, to obtain the services of suitable nurses for isolation hospitals, at short notice. This difficulty may arise in a county, at a time when the majority of its fever hospitals may employ a considerable number of nurses, who have no patients, and whose services could be advantageously utilised, for the isolation hospital or hospitals in other parts of the county, where cases demanded skilled nursing.

This fact has been recognised, and the scheme if adopted will, it is hoped, overcome most or all of the existing difficulties. A register for the county of Gloucester and the cities associated with it, and for the county and city of Worcester, respectively, will be kept by the respective county councils. All the hospital authorities in the two counties have been invited to approve and adopt this proposal, and this they have readily done, with the exception of Evesham, where arrangements have been made with a nursing association. It is hoped that, as a matter of discipline and economy, Evesham will ultimately reconsider its decision and join the scheme. Each isolation hospital authority participating, who (1) has nurses available, or (2) requires nurses, will communicate with the centre fixed for their area, giving certain necessary particulars. The object of dividing the counties into areas is for facility of communication, and to enable each centre to satisfy the needs of its area, to the fullest extent, before applying to another centre for nurses.

The scheme is well thought out in its details, and seems to provide against the difficulties which can be foreseen or anticipated at the outset. We shall hope to publish the rules so soon as they are finally approved. Meanwhile, it may interest our readers to know that the nurses, who may be sent from one hospital to another for temporary service, are afforded every protection, and that each will receive, in addition to her salary, one-third of the fee paid for her services, together with travelling expenses, to compensate her for moving temporarily from one hospital to another. As the regulations have been drawn up by the county medical officers, every care has been taken to avoid any risk of conveying infection through the nurse.

### The Care and Nursing of the Insane.

By Percy J. Baily, M.B., C.M.Edin., Medical Superintendent of Hanwell Asylum.

### II.—NURSING THE SICK.

(*Continued from page* 57.)

*Sick-room Appliances.*

(1) *Hot-water bottles.*—These are made of earthenware, tin, or india-rubber, the latter being in the form of bags. Whenever patients suffer from cold feet, as very many do, especially old people, they should be provided with some sort of hot-water bottle. Cold feet are often responsible for sleeplessness and restlessness, and the presence of a hot bottle may have a very soothing and quieting effect upon the patient. Whatever form of bottle be used it should be very carefully and completely covered with several layers of flannel or old blanket. When the patient is suffering from any form of paralysis, or if his vitality is lowered by prolonged disease or old age, great care must be taken that the bottle is not too hot, for a degree of heat which an ordinary healthy person could quite easily bear will in such cases often cause extensive burns on account of the bad nutrition of the patient's tissues. Also if the patient should be in an unconscious or semi-conscious state, or should be demented or in a state of anæsthesia after operation, special care must be taken to avoid burning him. The nurse should see that the heat is not too great by placing her own face against the flannel-covered bottle.

(2) *Cradles.*—There are many painful conditions which render the weight of the bed-clothes too much for the patient to bear. In these cases the bed-clothes should be held away from the affected part by the use of cradles. The most handy ones are those made of wicker work.

(3) *Water and Air Cushions.*—The most serviceable of these are either horse-shoe shaped or ring-shaped. They are made of india-rubber and should not be over filled. They are used to relieve pressure locally in parts that are threatening to become bed-sore. As a rule it is much more satisfactory to place the patient upon a water-bed.

(4) *Bed-rests and Bed-tables.*—When patients are convalescing from acute diseases and often during the course of chronic illness it is convenient at meal times, and is a welcome change at other times for the patient to be propped up in bed. This may be done either by placing pillows behind the patient's back or by means of a bed-rest, which is less liable to become deranged than pillows. A very good bed-rest may be improvised by turning an ordinary cane chair upside down behind the patient with a pillow over it for him to rest upon. Bed-tables are made with the support at one end so that the table may project over the bed. These add greatly to the comfort of the patient, who, although unable to get up is yet strong enough to sit up in bed. In many cases of heart disease some sort of bed-rest is essential as the patient may not be able to breath unless propped up.

(5) *Feeders.*—In a good many cases it may be inexpedient to raise the patient in order to feed him and then a feeder becomes necessary. These are merely cups which are partially covered over and which are provided with a spout. By means of such a feeder the patient is enabled to drink without raising his head from the pillow. A small piece of india-rubber tubing attached to the spout may render the feeding still less difficult.

(6) *Spittoons.*—These are necessary when there is any expectoration. They require to be frequently removed, emptied, and scalded. For ordinary cases of pneumonia or bronchitis the most convenient spittoon is one which is shaped like a mug with a funnel-shaped covering over it. When the sputum contains some specific organism as in phthisis (tubercle bacillus) it requires to be dealt with in a special manner in order to ensure destruction of the organism. The best form of spittoon for such cases is an open shallow tin or earthenware vessel which can be partially filled with sawdust. Each case should be provided with such a spittoon. The spittoon should be changed at least every four or six hours or more often if the sputum be abundant. The used spittoon is to be carried by the nurse direct from the patient's bedside to a fire and there the sawdust and sputum are to be burned. The tin or earthenware vessel is then to be thoroughly scalded with boiling water and afterwards dried and refilled with sawdust. Many phthisical patients are not confined to bed, for these various forms of pocket spittoons are made. These require very careful cleansing and attention, but the patient himself is generally able to look after this if he have an ordinary degree of intelligence. It is obvious, however, that such forms of apparatus can seldom be made use of in asylums.

(7) *Bed-pans.*—The use of the bed-pan is at first almost always strongly objected to by the patient, but in many cases of illness it is impossible either from the nature of the disease or from the extreme weakness of the patient, to get him out of bed. In these cases the bed-pan is an essential. There are two chief varieties in shape—namely, the circular bed-pan and the slipper—probably the latter shape or some modification of it is the most suitable and the least uncomfortable for the patient. Very great care must be exercised by the nurse when using the pan. It should be warmed before it is given to the patient, and that portion which must come into contact with his skin should have some sort of covering either of flannel or lint or a pneumatic pad (MacMillan's) may be used, the latter being both comfortable and easily kept clean. A small quantity of some disinfectant solution may be placed in the pan before use unless the urine or stool is required for medical examination. On no account must the bed-pan be pushed under the patient, this would inevitably tend to cause bruising and chafing of the skin which would be sure to end in bed-sore. If the patient is strong enough he may raise his buttocks from the bed himself while the nurse places the pan in position, but if he should be too weak to do this he must be raised up from the bed by the nurse and the pan placed beneath his buttocks by an assistant. The best way to raise the patient is to place the forearm beneath his bent knees and the other hand under the upper part of his sacrum. When the pan has been placed in position he is to be gently lowered on to it. When the pan has been used it should be withdrawn in a similar way and at once covered with a damp cloth and removed from the patient's vicinity. If it contain an evacuation from the bowel, this should always be disinfected as described later, at all events if the patient is suffering from any condition where the stools are likely to contain infective organisms as in typhoid, dysentery, the diarrhœa of phthisis, etc., if not in every instance. In dealing with male patients the urine should be received in special bottles for that purpose.

When the pan has been removed from the patient he requires to be carefully attended to. The anal orifice should be wiped and dried with pledgets of cotton-wool, very particular care being bestowed upon those portions of the buttocks which are opposed to and come into contact with one another: these parts should be dusted with powdered boric acid.

### Ventilation.

The air of any room or other enclosed space (theatres, churches, etc.) in which there are human beings is constantly being vitiated or polluted, as we already know, by the exhalations and emanations from the lungs and bodies of these individuals. By the term "ventilation" is meant the removal of the foul air thus produced and the substitution for it of pure fresh air from the outside. The chief difficulty is to obtain this result without the production of appreciable draughts.

Vitiation of air by human beings. In the account of the physiology of the respiratory system the composition of the atmosphere has already been indicated and we have seen that the breath of human beings differs from pure fresh air in many important particulars. It contains about one hundred times as much carbonic acid as fresh air and about one-fifth less oxygen. It also contains a largely increased amount of moisture and is hotter than the surrounding air. In addition it contains suspended in it a certain amount of organic matter which consists of minute and, of course, invisible particles of food from the mouth as well as some of the cells from the superficial layer of the mucous membrane which lines the air passages. These minute particles of organic matter are brushed off and carried away by the air current as it passes to and from the lungs. This organic matter in suspension rapidly undergoes putrefaction and becomes highly poisonous, its presence is indicated by the stuffy close smell which is peculiar to a badly ventilated room. This peculiar sour and oppressive smell is at once noticed when one passes from the outside fresh air into the badly ventilated room, but the sense of smell rapidly becomes accustomed to it, so that after being in such an atmosphere for even a short time it is no longer appreciable.

But it is not only from the lungs that the air of an inhabited room receives polution. A large amount of moisture (sweat) is given off by the skin which evaporates into the atmosphere and from the skin also comes a considerable amount of organic matter in the shape of epithelial cells which are continually being brushed off the superficial layers of the cuticle.

All these may be said to be normal sources of atmospheric pollution, but when dealing with sick people others have also to be added such as the discharges from wounds (ulcers, bedsores, etc.), and when the patients are of faulty habits the evacuations from the bowel, urine, etc.

Artificial light (gas burners, candles, lamps) add largely to the amount of carbonic acid and other impurities thrown into the air: an ordinary gas burner will produce as much carbonic acid as two or three adults. The amount of carbonic acid may, however, be very largely increased without serious risk to health, the danger of inhaling the air of ill-ventilated places being chiefly due to the organic impurities it contains.

Effects of breathing vitiated air on health. People who habitually live in badly ventilated rooms become generally pale and sickly. They are anæmic and suffer from headaches, giddiness, and lassitude. In addition to these less serious effects it is now universally recognised that bad ventilation is a potent factor among the predisposing causes of consumption and other tuberculous diseases.

*(To be continued.)*

### The Nurses' Clinic.

### RESTLESSNESS.

Restlessness may be a sign of exuberant life and vital energy, as shown in the active, healthy child, to whom sitting still seems a physical impossibility; it may be simply one of the inevitable characteristics of a nervous temperament, evidenced in the adult in a number of fidgetty little ways, causeless anxieties, or irritating mannerisms. It may be a prominent symptom of disease, as the purposeless movements of chorea, and it may exist as the precursor of delirium tremens, of insanity, and some forms of brain and nerve trouble.

Apart from this, it is a very common feature in most cases of illness. Restless, disturbed sleep precedes and accompanies many of the infectious complaints and various acute forms of disease. It is a bad sign in typhoid fever, and after an abdominal operation. In the last stage of phthisis or heart disease increasing restlessness usually marks the beginning of the end.

It is very necessary that a nurse who has to do with a restless patient should herself be calm, and of an even temper and disposition. One who fusses and fidgets about the room or ward, does all her thinking "outside her head" in the form of ceaseless chatter, shows her anxiety over a patient, or her worry about the work by a perpetual frown or a pre-occupied look, has not the same calming influence on a sick person as one who by grace of movement, gentle speech, and quiet manner reassures those under her care, and causes them to rely on her as on someone stronger than themselves.

When restlessness is present there is always some cause for it. This the nurse should at once seek to find out and remedy if possible.

In an infant it may be due to hunger or thirst, heat or cold; the child may be wet or soiled; its clothes or bed may not be comfortable; a pin may be sticking into it;

THE NURSES' CLINIC.—*Continued.*

abdominal pain, caused by indigestion, may be present; acute brain or chest trouble may be threatening; or one of the specific infectious diseases about to develop.

It is always a safe plan completely to undress an infant where the cause of restlessness cannot be otherwise ascertained, and a warm bath will often do wonders in alleviating its distress from whatever cause, and inducing sleep.

In older children restlessness may be due to the shock consequent upon an accident or operation : if in a hospital, to home-sickness, or fear of the dark, discomfort caused by having to lie in a constrained and unusual position; or splints and dressings may be giving pain.

In adults the causes are almost too numerous to mention, and need much careful treatment on the part of the physician. An intelligent nurse can be in such a case of the greatest aid, seeing, as she does, so much more of the patient than the doctor, and having so many more opportunities of gaining his confidence.

The more obvious causes of restlessness should first engage the nurse's attention, and not until they have all in turn been satisfactorily disposed of should she attempt to discover those that may be more remote. She should see if the patient's bed, clothing or dressings are comfortable and in place. She may remove or add an extra blanket or hot bottle as may be required, shake his pillows, offer food or drink, see if the bowels or bladder need attention, and whether any noise, movement, or too strong a light is annoying him. If the cause is mental anxiety a patient will often give his confidence to a sympathetic, discreet nurse, who may be able to soothe him by offering to write or send an urgent letter or telegram, or communicate some wish to an absent friend. Sponging the face and hands, brushing and rearranging the hair, altering the patient's position in bed, if helpless, or the rubbing of paralysed limbs, will sometimes help in soothing restlessness, and if combined with a suitable hypnotic, may work wonders. The sleeping draught should always be given with a brightly-expressed belief in its efficacy by the nurse. The weaker mind of the sick person, clouded with disease, will instinctively rely on the assurance of the stronger, that the medicine will make him sleep, and the restlessness will thereby be dispelled.

Rest is so essential a law in the process of healing, and so vital a principle of good nursing, that every effort should be made to secure it for the patient's recovery.

### Incidents in a Nurse's Life.

### FIRST NIGHT IN A LYING-IN HOSPITAL.

It was my first night in the Lying-in Hospital, and I was preparing for bed, feeling homesick and full of dread lest I should be called up in the night. Being my first case, I was convinced in my own mind that in my nervousness I should do just what I ought not to do.

The night portress suddenly banged into my room in her usual unceremonious fashion with, " A case for you, nurse."

I sallied forth with a sinking heart and met the midwife on the stairs.

" Are you Nurse E.? " she inquired. " Yes." " Ever done nursing before? " " No." " Then you will find it all very strange, nurse; but you will soon get used to it. Come along."

I went along, and earnestly listened to her explicit instructions on the way.

When we entered the squalid room we heard groans and moans, not only from the patient but from her numerous friends. The midwife tactfully got rid of them all but one—the mother, a stout old lady with a bad " heart," to which she incessantly referred!

I admired the way in which the midwife managed the necessary arrangements, and still more the way in which she managed me. So cleverly was this done that no one could have imagined that I was a timid novice. She gave me such clear directions that I got on fairly well; but we were very short of hot water, and when the old lady with the " heart " was appealed to, she declared that afflicted organ forbade her rushing up and down the stairs, but if I would go into the first house on the right I would find a kettle of boiling water which her cousin had promised to get ready. " Just walk straight in, nurse; she knows you are coming." I walked straight in, and found the kitchen full of men playing cards. I smiled politely on them and bore off the kettle. I was only just back in time. Five minutes after I got into the room baby arrived—a fine strong specimen. The mother, who had been very brave, waxed most eloquent, and wished to embrace us both.

I had never had anything to do with little babies, and when the midwife said, in her cheerful matter-of-fact way, " Now, nurse, you must bath the baby," my heart sank. I shall never forget that performance. The baby was so slippery, and I was so incompetent. I tried to make a lap for it, and felt quite injured when the oft-repeated " knees together, nurse," came. It got finished at last, somehow, then we cleared up, and I sallied forth to return the kettle. I remarked to the old lady, now violently fanning herself with a newspaper and foretelling all kinds of prospective heart spasms in the night, " What a number of men there are in the kitchen next door."

" Men? There are no men there, only Liza's old man."

" Well, I saw about a dozen playing cards."

" Law! Wherever did you go? "

" Where you told me, first house on the right."

" Mercy on us, did I say the first house on the right. I meant the left! "

I went back, knocked this time, apologised, and explained the mistake. They were all very nice about it; but whatever they must have thought of my calm audacity in bearing off their kettle from their fire without a word of apology I do not dare to think.

We returned to the home, as I fully believed to bed; but to my dismay I was sent to two more cases that night. The second frightened me considerably, as we had to summon the doctor, who put on forceps; but by the time the third was over I made up my mind to persevere and to win the coveted certificate of the Central Midwives Board, which I rejoice to say is now in my possession.

### Death in our Ranks.

The remains of " Sister York " (Miss Murphy), who was trained at the Sussex County Hospital, Brighton, subsequently becoming sister in the same institution, were laid to rest on Friday last at Worthing. Miss Murphy recently contracted infection while engaged in the performance of her duties, and succumbed to the disease at the early age of thirty-one. A memorial service, held in the hospital chapel on Wednesday, October 31, was attended by a number of patients and nurses. " Sister York " was very popular with both, and was devoted to her work.

### The Alleviation of Suffering from Hernia.

### EXAMINATION QUESTIONS FOR NURSES.

The question was as follows : "In the case of inguinal or femoral hernia (in private or district work) what steps should you take to alleviate suffering pending the arrival of the doctor?"

### First Prize.

I should get the patient into bed, placing a pillow underneath the knees to relieve the abdominal muscles. If the patient had been vomiting he would most probably be very cold and collapsed, in which case I should apply hot blankets and hot-water bottles, or a substitute for them, such as bricks made hot in the oven, hot flat-irons, or even hot dinner plates wrapped in flannel, or any ordinary bottle, with a good safe cork, filled with hot water and treated in the same way.

If the patient were vomiting or had a cough, I should support the hernia with the hand, applying gentle pressure each time the patient coughed or vomited.

If the hernia were very painful, I should apply flannels wrung out of hot water, which would not only relieve pain, but help to reduce the inflammation and swelling, so that the doctor might be able to reduce the hernia by manipulation.

I should keep the mouth moist by moistening the lips frequently, but not allow the patient to take any food before the doctor's arrival.

I should also get ready for operation, for if the hernia were strangulated this would probably be done without delay.—"Central."

### Second Prize.

Place patient in bed, keep knees drawn up by placing a pillow or cushion under knees, then apply ice, broken in small pieces, placed in a waterproof bag—a sponge bag will do—or tied up in a piece of jaconet or any thin waterproof material, over hernia.

If ice is not procurable, apply flannels wrung out of hot water over hernia, or place patient in a hot bath in a recumbent position, with legs drawn up as in bed. Keep patient as quiet as possible. Great care to be taken when placing in bath or in any movement, so as not to cause strangulation.—"Phœbe."

### The Prize Winners.

"Central" takes the first prize and "Phœbe" the second, because their papers are the best all round, though they have failed to notice some points mentioned by other competitors. The papers this month are, on the whole, good; but there is none that stands out very markedly as evidence of ability. The most serious defect is that candidates do not differentiate between the treatment of the three kinds of hernia—reducible, irreducible, and strangulated.

### Honourable Mention.

This is gained by six competitors, who all send fairly good answers, subject to the omission of not recognising sufficiently that different cases require different treatment. "New Cross," "Hygeia," "Mirror Reader," "Elsie," "Nurse" (a very impotent and ineffectual pseudonym), and "Dot" all receive honourable mention.

### Question for November.

This will be the same as last month, but with an addition, which I hope will be thought out carefully by candidates, as it is of the utmost importance. The answers sent in this month show that competitors have not realised the serious nature of the issues depending on a correct appreciation of the situation involved. Question : In the case of inguinal or femoral hernia (in private or district work) what steps should you take to alleviate suffering pending the arrival of the doctor? Differentiate in your answer between the necessary treatment that should be tried for the three kinds of hernia—reducible, irreducible, and strangulated, and remember to keep strictly to your own work, and not to usurp that of the doctor.

The Examiner.

### St. Mary's Hospital, Paddington.

### PRESENTATION TO MISS MEDILL.

The Board-room at St. Mary's Hospital, Paddington, was filled on Thursday last week, the occasion being the presentation of a testimonial to Miss Medill, who recently resigned the post of matron. Among those present there were Lady Edward Spencer Churchill, Mr. J. R. and Mrs. Mellor, Captain and Lady Cecilia Webbe, Major-General Shaw-Stewart, Drs. Sydney Phillips, Bird, Rendel, and Feloe, and Mr. G. A. Byron and a great number of nurses.

Mr. J. R. Mellor, who was in the chair, read a letter from their chairman, Mr. H. A. Harben, stating his regret that the state of his health did not permit him to attend, and expressing his high sense of Miss Medill's loyal and devoted services to St. Mary's. He announced that the Central Branch of the Ladies' Association had now collected the sum of £500, which completed the endowment of a cot. Miss Medill and the sisters of the hospital, past and present, had taken great interest in the work of the Association, and had rendered much help in collecting this sum. Mr. Mellor went on to say that they were met there to do honour to a lady who for twenty-one years had satisfactorily performed most important and onerous duties.

Captain Webbe, treasurer of the Presentation Fund, said he was the oldest member of the House and Finance Committee, and had taken an active part in the work of the hospital for twenty years, and during all that long period he had never had anything but most cordial relations with her. During all this period Miss Medill had never asked the Committee for anything for herself. Owing to the increase in the staff, material improvements in the conditions under which they worked had been possible : they had more days off and more time for holidays. The dietary of the nurses had been improved, and they also had the satisfaction of seeing that their staff were now adequately housed. He wished to allude to the loss the hospital had sustained through the departure of Sister Colborn, and express the great appreciation of the Board at the inestimable services she had rendered for nearly eighteen years in various capacities. He would now ask Miss Medill to receive from the nursing staff, the Board of Management, the surgical and medical staff, and many other friends, a silver bowl and a cheque contained in the purse.

Mr. G. A. Byron then said that the sisters and nurses, past and present, who had been bound to Miss Medill with such close ties of relationship, wished that relationship to be commemorated by a gift in which no one else should have part or share—one that should be entirely their own. They therefore presented her with two albums, one containing their photographs and the other their autographs. The photographs of the chaplain, the medical superintendent, and the secretary had been included in the album.

Miss Medill said she had never wished so much before for the tongue of a ready speaker. She could not lay claim to all that had been so kindly said of her work. After all she had only tried to do her duty. She felt to the very heart all the kind and friendly feeling that was involved in the presentation. To the Board, who had always been so kind and generous, to the Committee, to the members of the staff, who had always been kind and considerate to her, to her good colleagues, and to her dear, dear nurses, and to all her many kind friends she wished to render all the gratitude she possessed. She trusted that they would all go on working for many years, doing good work in different ways; and, as they were all journeying towards the great goal, she would not speak of separation, but would say "Adieu."

When the applause which followed the conclusion of Miss Medill's speech terminated, she made a tour of the crowded room in order to thank everyone individually for their share in her presentation.

The massive "Cashel" bowl is of solid silver, bearing the following inscription: "Presented, with a Purse of Gold, to Miss E. M. Medill by Past and Present Nurses and Members of the Board and Medical Staff on her retirement from the post of Matron of St. Mary's Hospital, October 1906." The two albums, handsomely bound, bear a monogram. The cheque was for £280.

### Meeting of the Central Midwives Board.

A MEETING of the Central Midwives Board was held at Caxton House, Westminster, on Thursday last week, Dr. Champneys presiding. Those also present were Miss Wilson, Miss Paget, Dr. Dakin, Sir William Sinclair, Mr. Parker Young, and Mr. Ward Cousins.

### Restoration to the Register.

In compliance with a resolution of the Board last May the Clerk of the London County Council reported by letter on the conduct of two certified midwives who had brought on themselves the Board's censure for an infraction of the rules. The report was to the effect that both midwives had in the interval complied with the regulations and it was decided to reply to the Clerk of the London County Council expressing satisfaction at this result.

### Letter from the Jubilee Institute.

A communication, received on the day of meeting, was read from the Secretary of the Queen Victoria's Jubilee Institute for Nurses, enclosing a resolution which was passed by the Council on October 31:—

That in the opinion of the Council of the Queen Victoria's Jubilee Institute for Nurses it would place great difficulties in the way of the working of the Institute for the training and supervision of midwives to be in any measure withdrawn from the control of the Central Midwives Board, as would apparently be done by the exemption suggested in the amendments of the Privy Council to the Board's revised Rules.

The letter went on to state the reasons for the resolution, as follows:—

"That a large number of the Queen's nurses are drawn from women trained in Poor-law infirmaries, that training included midwifery training, so that when engaged for district work by the Institute they already hold the Central Midwives Board certificate. The question of the standard of the midwifery training given in these infirmaries is therefore one which nearly concerns the Institute.

"At the present time the Central Midwives Board certificate is a guarantee of sufficient training, as all must have attained the requirements of the Board before examination; but, under the proposed amendments, a second standard of training, beyond the control of the Central Midwives Board, would be established for candidates from Poor-law infirmaries, and the Board's certificate would, for such candidates, lose its greatest value.

"The Queen Victoria's Jubilee Institute would be unable to accept Poor-law midwives without further investigation as to their training. Incidentally, this limits the employment by the Institute of midwives trained under the Poor-law, and would, in consequence, entail an unnecessary hardship on a very desirable class of women. The Queen Victoria's Jubilee Institute has unequalled and practical opportunities for observing the working of the rules of the Central Midwives Board, which, they are able to say, have on the whole worked satisfactorily for the protection of the lying-in woman and the improvement of midwives."

The Council hoped that the Board would not adopt amendments creating a second standard of training.

It was decided to thank the Institute for its valuable communication.

### Re-opening of Cases.

Mr. Fordham had given notice to move a resolution with respect to the reconsideration of midwives' cases, and, on the motion of Mr. Parker Young, it was adopted in an amended form, to the following effect:—That where an application is made by or on behalf of a midwife, whose case has been definitely dealt with after citation and a hearing by the Board, for the reconsideration of such case, the Secretary do inform the applicant that the Board are not prepared to reopen cases in which they have given formal judgment, and do refer the applicant to the rules.

Some other business was transacted, and the Board adjourned.

### Glasgow's Interest in the National Pension Fund.

THERE was a great gathering of nurses at the Christian Institute, Bothwell Street, Glasgow, on the afternoon of Friday last, to hear an address on the aims and objects of the Royal National Pension Fund for Nurses by the secretary, Mr. Louis H. M. Dick.

The Lord Provost presided, and, in introducing the speaker, said that he had the greatest confidence in recommending the Pension Fund to the nurses of Glasgow, whose unselfish devotion to the sick they all appreciated, and he desired to add his tribute to the debt of gratitude which they all owed for services so well rendered.

Sir John Ure Primrose, Bart., supported the Chair, and fully endorsed the remarks of the Lord Provost, adding that he had gone carefully into the affairs of the fund, and could answer for its soundness and its stability.

After Mr. Dick had given his address, Professor Henry Clark, C.M.G., F.F.P.S., proposed a vote of thanks to the Chairman, and spoke of the great interest which he had always taken in the fund. He referred to the fact that the Victoria Infirmary paid for policies on behalf of those nurses who became members of the fund, an example which he hoped would be followed by every hospital in Glasgow.

Mr. George Green, J.P., inspector in Scotland for the Prudential Assurance Company, said he noticed amongst the pamphlets on the table one containing a statement by Mr. Dewey, the general manager of the "Prudential," that the fund was in a better position to give a better value in the shape of pensions for the premiums paid than any other commercial undertaking. Mr. Green wished his hearers to remember that Mr. Dewey's opinion on insurance matters carried the utmost weight, for no one was better qualified to advise on the subject. Speaking himself as an insurance man of some experience, he had been specially impressed by the extraordinarily low rate of the working expenses, which were much less than those of any ordinary insurance society. Amongst those present were Mrs. Strong, matron of the Royal Infirmary; Miss Macfarlane, matron of the Victoria Infirmary; Mrs. Pearce Campbell; Miss Moseley, matron of the Western District Hospital; Miss Wright, matron of the Stobhill Hospital, Springburn, and other matrons. On Saturday Mr. Dick addressed a meeting of nurses at the Royal Infirmary, Glasgow, by the invitation of Mrs. Strong, the matron.

### Everybody's Opinion.

[Correspondence on all subjects is invited, but we cannot in any way be responsible for the opinions expressed by our correspondents. No communication can be entertained if the name and address of the correspondent are not given as a guarantee of good faith, but not necessarily for publication. All correspondents should write on one side of the paper only.]

### THE STANDARD OF NURSING.

"A Sister" writes: Much is said nowadays about raising the standard of the nursing profession. But how can the standard be raised when such advertisements as this are seen? "Wanted, a fully-trained nurse to take charge of small hospital, whose husband will fulfil the duties of a porter." I myself am a trained nurse, and feel that it is an insult to trained nurses. How can we expect to be treated with anything like respect if this is the state of things? I have done both hospital and private work, and I must say that I and my nursing companions have always been treated as ladies, not as a porter's wife would expect to be treated. One begins to wonder what the nursing profession was like before the modern improvements towards making it a profession fit for ladies with education to enter. I write on behalf of many nursing friends, as well as on my own account.

### *Re* "BREACH OF CONTRACT" CASE.

Nurse Jukes writes: I have read the correspondence addressed to you by the Rev. R. Free, Miss Heatley, and others, and, curiously enough, the writers all appear to avoid the main questions at issue in the case—namely, (1) "What was the contract between Miss Heatley and myself?" and (2) "Did Miss Heatley commit a breach of the contract?" From the letter written by Mr. Free it is evident that he is not acquainted with the facts of the case, or he would not suggest that the judge's finding was "an apparent miscarriage of justice"; and for him to say that the judge's decision entitles an applicant at the defendant's home to disobey the rules of the establishment and demand her money back is absurd, and is a further proof of his ignorance of the matter. Had he been present at the court and heard the evidence he would not have troubled you with any comments on the case. The facts shortly are these, and they are substantially borne out by the correspondence which passed between myself and Miss Heatley. In May the defendant, knowing I wished to enter for the October C.M.B. examination, agreed to give me three months' tuition for £16 16s., which would enable me to qualify for the examination. I started at the home on June 25, and a few days after I paid the defendant £15 15s., making with the £1 1s. already paid as entrance fee the sum of £16 16s., for which Miss Heatley gave me a receipt "for three months' training in midwifery." Observe that the receipt is not "on account." In face of this Miss Heatley suggested that I agreed to pay her £25 for four months' training, and at the end of my first month demanded a further sum to make up the £25, and refused to give me any more cases unless I paid her, and, in the alternative, said "that I could go." I was obliged to adopt the latter course. In view of these facts, it is to be wondered that the judge decided that the contract was as above stated, and that defendant had committed a breach of it in refusing to give me the training unless I paid her further fees? As the correspondence published by you makes it appear that I was in the wrong, notwithstanding the decision of the judge, I trust that you will allow this explanatory letter to appear in your columns.

### THE NURSES' HOSTEL.

"A Contented One" writes:—The malicious letters in your last issue against the hostel and its management oblige me to write in its defence. I have lived at the hostel—South Block—between my cases some years, and cannot speak too highly in its favour. I have always received the greatest courtesy from the management, and am sorry that they have been subject to the unjust attacks made by some unscrupulous and dissatisfied women among our number. This opinion is endorsed by the majority of reliable nurses who make the hostel their home between their cases. The proof of my statement is in the fact that both blocks are continually full.

"A Hostel Nurse" writes:—Before this correspondence ceases I should like to give a simple correct idea of life at the Nurses' Hostel, where I have lived for some years in both North and South Blocks. Each nurse pays 17s. 6d. a week in a cubicle or double room and 25s. in a single room, which includes board; there are no extras. Tenants, who furnish their own rooms, beside rent, which varies from 8s. to 11s. 6d. a week, pay 10s. 6d. a week for board if they take their meals in the hostel, which is optional. The house is kept scrupulously clean, the food plain, but varied as much as possible, and well cooked; the meals are very punctual. I have always found the heads courteous, and I consider that for the terms we are liberally treated in every way but one. I always feel grieved that the telephone is not answered at night; doctors arrive at a patient's house, and are obliged to remain there until a trained nurse is obtained. Many of their own nurses reside at the hostel; if they could telephone and have one sent at once they would gladly do so; failing the telephone there they apply elsewhere, and we lose much work. The new management has no fair chance of showing what it can and will do while this dissatisfied, unreasonable spirit is encouraged among "the many" by the few apparently would-be stump-orator nurses, who certainly are not of a disposition suitable for their profession.

"One Who Signed the Petition" writes: Before obtaining my present appointment I was private nursing in London for three years, putting up at the Hostel between my cases, and know it well. The discontent there has been simmering ever since I knew it, nurses complaining bitterly of rudeness by officials of the hostel answering the call to doctors and others telephoning to them on business matters, and loss of work resulting therefrom, also general discourtesy, indifference to visitors, and many discomforts. If you ventured to complain you were told you could always go elsewhere, and the cheapness was quoted. Personally I never thought it cheap. 17s. 6d. a week meant sharing a room with another, the room often scarcely large enough for one; messages, baths, boots, boxes, cupboards being extras, and a certain amount of tipping is expected. When the new superintendent arrived we hoped for reforms, with what results is already known. The only charge against Miss Hulme is that she wanted to turn it into a nurses' institution. Supposing that this was so, it had been done for years, many residents in the district applying there for a nurse; and these were sent without any inquiry made as to their training and credentials. Miss Hulme would at least have made sure the nurse sent out was a trained one. The hostel will only improve when it has another to compete with.

"M. A. B." writes: I should like to give my one and only experience of the Nurses' Hostel. I wanted a bed and breakfast previous to going to an early operation the following morning. I telephoned to the hostel, and was told that I could have a bed, but that no breakfasts were served before 8 a.m. As I had to be at my case by 8.15 a.m., and had a fifteen minutes' drive, I decided to take in something from a confectioner's to stay the pangs of hunger. I was going to spend the evening with friends, so I took my bag to Francis Street early in the evening, and wished to settle my account then, as I was leaving early in the morning. I made some slight demur at finding I was to be charged for the breakfast of which I should be unable to partake. So, after some hesitation, the Secretary (I believe) said she would have some bread-and-butter and a cup of cocoa sent to my room overnight, and I could heat the latter up on the gas stove. Having learned in my training, amongst other useful things, to be thankful for small mercies, I went to my friends', and returning at a few minutes to eleven, found the door locked and another nurse waiting on the door-step. We rang, and presently the door was opened by a lady (I know now she was Miss Wood), who expressed herself in decided tones of annoyance. We mildly suggested that it was not yet eleven o'clock, and were told to "Look at the clock," which we did; it was just two minutes to the hour. I made some comment to my companion as we mounted the stairs. Miss Wood said: "No talking as you go up-stairs, please," or words to that effect, in the tone in which one would imagine a cook might be greeted on coming in late. I found a cup of very anæmic cocoa and two very thick slices of bread-and-butter awaiting me—my breakfast, for which I had paid the sum of 9d. (or is it 1s.?), and somehow I felt that I had had a favour bestowed upon me. The idea seems to be to run the hostel as much as possible on hospital lines, which is the last thing a nurse would desire. When she is once trained and earning her own living she is an independent individual, and rightly expects to be treated as such, seeing that she is prepared to pay for what she has. I hope we may yet see the "ideal hostel" so ably described in your columns.

"A Victim" writes: In consequence of the "evictions" at the Nurses' Hostel many of us wondered who would be the next, and several who had signed the petition decided to write to the Board, as Miss Chamberlain (the new lady superintendent) was unable to give us any satisfactory information. We were informed that a Board meeting was to be held at the end of last week or beginning of this, so I wrote the following letter to General Sir Allen Johnson on Miss Chamberlain's advice:—"Dear Sir,—With reference to the recent refusal of the Manageress of this Block (South) to admit two nurses on their return from cases without due notice, and while beds were disengaged, I should be glad to know whether or not I am to expect the same treatment on my return after my next case, to which I may be called at any moment. This address has been my headquarters for the last four years, and is known to patients, doctors and friends, here and abroad; therefore, to be turned from the door on my arrival might lead to unnecessary loss of work and other complications, not to mention the difficulty of finding suitable accommodation at such short notice. The feeling of uncertainty in the minds of many of us is great, therefore a reply at your earliest convenience will greatly oblige." The meeting was held last Saturday. I had in the meantime gone to a case for a few days. After the meeting I went to Miss Chamberlain and asked for my answer, but was informed that the Secretary would give it me; on asking for her I was told that she was away. I then asked when I was to hear the result, as I expected to return on Monday, and did not wish to be turned from the door, and I was promised I should hear on Monday. On Sunday I asked Miss Edwards at the South Block for a bed. As there was not one at liberty I was promised one for Monday. I arranged to stay another night with my patient, but on my arrival at the South Block on Monday morning I was informed that there was no bed at liberty, and a note was handed me from the Secretary stating that in future I must find accommodation elsewhere. A nurse who was leaving had on Sunday given me leave to mention the fact to Miss Edwards, which I had done, but was still refused it. After the Board meeting Miss Chamberlain and Miss Edwards had a consultation of over an hour, so perhaps further comment is needless. I hope that you may be able to make the contents of this letter public.

[This correspondence must now cease.—Ed. Hospital.

### Novelties for Nurses.

(By our Shopping Correspondent.)

### THE NURSES' SALOON.

We are asked by Messrs. E. and R. Garrould, 150 to 160 Edgware Road, to state that the width of their new belts alluded to in the notice in our ossue of October 27, under the heading of "The Nurses' Saloon," is 3 inches, and the price 8½d., not 3½d.

### Appointments.

Addenbrooke's Hospital, Cambridge.—Miss Margaret F. Macintyre has been appointed sister of the Out-patient and Massage Department. She was trained at the Glasgow Royal Infirmary, and has since been sister-in-charge at the Consumption Sanatorium, Bridge of Weir, N.B., and private nurse and masseuse in connection with the Kent Nursing Institution. She holds a certificate for massage.

Bromsgrove Isolation Hospital.—Miss Amy Robinson has been appointed staff nurse. She was trained at St. George's Hospital, London, and Brighouse Isolation Hospital. She has since been staff nurse at the Corbett Hospital, Stourbridge, and district nurse at Middlesbrough.

Children's Cottage by the Sea, Queenscliff, Australia.—Miss Mabel Chinn has been appointed matron. She was trained at the Alfred Hospital, Sydney. She has since been sister at Geelong Hospital, and she has done private nursing in Melbourne for ten years.

Crumpsall Infirmary, Manchester.—Miss Lucy Bentley has been appointed night superintendent, and Miss Pleasance Aurelia Hall ward sister. Miss Bentley was trained at Crumpsall Infirmary, and has since held the post of ward sister for nearly three years. She is registered under the Central Midwives Board. Miss Hall was trained at Crumpsall Infirmary.

Droghera Memorial Hospital, The Curragh.—Miss Agatha J. Cullen has been appointed nurse-matron. She was trained at the Westminster Hospital, London, and has since done private nursing in Dublin.

East London Nursing Society.—Miss Helen A. E. Taylor has been appointed superintendent of the Central Division of this Society, which is affiliated to Queen Victoria's Jubilee Institute for Nurses. She was trained at St. Thomas's Hospital, London (where she was subsequently in charge of the infectious block), and at the Rotunda Hospital, Dublin. She received her district training at the Shoreditch Home, and she has since worked as Queen's Nurse at Faringdon, Berks, and as senior nurse at Kettering.

Hastings, St. Leonards and East Sussex Hospital.—Miss Gertrude A. Heinrich has been appointed matron. She was trained at Warminster Cottage Hospital and St. Thomas's Hospital, London, and has since been sister at the Isolation Hospital, Bromsgrove, and matron of the Throat and Ear Hospital, Brighton. She has also taken temporary sister's duties at the Children's Hospital, Shadwell, London.

Isolation Hospital, Warrington.—Miss F. Fairbrother has been appointed staff nurse. She was trained at Hampstead General Hospital, and has since been staff nurse at the Gordon Hospital, London, and assistant nurse at the Fever Hospital, Faversham. She has also done district and private nursing.

Metropolitan Asylums Board.—Miss Fannie Judge and Miss Florence Green have been appointed charge nurses under the Metropolitan Asylums Board. They were both trained at St. Mary (Islington) Infirmary, and have since been staff nurses under the Asylums Board.

Perth District Asylum, Murtal.—Miss Agnes Baird has been appointed charge nurse. She was trained at Gartloch Asylum and the C.C. Hospital, Irvine. She has since been charge nurse at Gartloch Asylum, has acted as night matron on holiday duty in the same institution, and as matron, for a short time, of the C.C. Hospital, Irvine. She has had massage and maternity training.

Victorian Infant Asylum, Australia.—Miss Conolly has been appointed lady superintendent. She was trained at the Children's Hospital, Melbourne, and has since been staff nurse at Dr. Moore's Private Hospital.

West of England Eye Infirmary.—Miss Rose M. Penny has been appointed matron. She was trained at the West of England Eye Infirmary, Exeter.

### A Book and its Story.

### BENEFITS FORGOT.*

Mr. Bloundelle Burton has taken for the background of his seventeenth-century romance the conspiracy which was devised in France to remove Louis XIV. from the throne and to place in his stead Louis, Prince and Chevalier de Beaurepaire. The peculiar infamy attached to the design lay in the fact that Prince de Beaurepaire had been the chosen playmate and companion of the king in childhood, and was, at this period, the Colonel of all the Royal regiments of guards. The plot, spoken of as "the great attempt," had its origin in Normandy, and was only one of many which had for their object the deposition of Louis XIV., who, by his aggressive policy and the imposition of heavy taxes, had made himself unpopular with the people. He had enemies abroad as well as at home, Spain being among the countries who regarded him with feelings of enmity. The inhabitants of three of his own most important provinces were favourable to the rising, and assistance in money, arms, and men had been promised by Spain. The ostensible cause of the plot was the imposition of a tax on woods and forests. Normandy abounded in forests, and the timber in them formed a considerable source of income to the land-owners. Taxes had for centuries existed and were submitted to by the Normans, but the imposition of a new one was the cause of covert discontent which by degrees grew into the plot which should cut short the already long reign of Louis XIV. Wealthy landowners of Brittany and Guienne took part in it. How far-reaching were the designs of the promoters may be judged from the following passage:

"Louis had earlier than this deprived Spain of some of her most cherished possessions, and it was now suggested to the Governor of Brussels, that, if his country were willing to supply the Norman conspirators with money, arms, and men, Quillebeuf, at the mouth of the Seine, in the Bay of La Havre, might be seized by a hostile fleet. And, since half the country between that place and Paris would be favourable to the designs of the invaders, six hundred men well mounted and equipped could easily reach Versailles, overpower the detachments of regiments serving there as the King's Guard, and not only possess themselves of his person, but also of the person of all the Royal Family. A republic such as that of Venice or Holland was to be founded, de Beaurepaire was to be the President, and ample funds to be supplied by Spain."

A vivid picture of King Louis and his court at Fontainebleau is given: "The splendid one—'La Dieudonné'—Louis XIV., King of France and Navarre, sat in the *Galerie des Serfs* at Fontainebleau, before a blazing log fire, his feet and legs encased in long heavy riding boots, half a dozen dogs around him, and, on his lap, a little spaniel of the breed afterwards known in England as that of King Charles, with whose long silky ears he toyed. Near the King, yet still at some distance from him, were many members of his family and court, including the Queen, who sat before a second fire further down the room, in the riding-dress in which she had that day accompanied her husband to a wild-stag hunt in the forest. A little distance off, chattering, laughing, in discreetly subdued tones, were women who bore, or were yet to bear, names the world will never forget." The King had before him a small table on which was piled up in disarray a vast heap of letters that had arrived that afternoon by special courier. He was reading those handed to him—after having first perused the contents—by the Marquis de Ludovois, his Minister of War. A Minister who had for years been gradually gaining an ascendency of the King, and between whom and Madame de Maintenon keen rivalry existed as to which influence should be paramount. Among the letters that specially arrested the King's attention were two, both of the same import. These he placed faced downwards and together on an accumulated pile on the table: "Two letters, from two women, and each telling the same story. Letters coming, you observe, from widely different cities. One from London, one from Geneva. Almost it seems there must be some truth in what they tell." The King addresses the Marquis de Ludovois, after some comments on the writers and their ability to acquire correct information on the subject of the conspiracy. Then, lowering his voice, and changing the subject, he asks: "Is he gone?" "He is, Sire, in this room." "Summon him." De Ludovois runs to obey the order and proceeds down the gallery until he reaches de Beaurepaire, who is talking to a lady, Madame de Sévigné; "the lady possessed a weak, simpering face that, in her case, was no index to her mind, and whose little curls all over her head gave an appearance of youth to which she had no longer any claim." The King, with the knowledge of the plot revealed in the letters, receives de Beaurepaire as a loyal subject. He tells him of enemies by which he is surrounded and appeals to him by the memories of the happy days when as children they had been playmates and comrades up to the present day when he expresses his satisfaction at the way he carries out the command of Colonel of his regiment of guards. The King continues: "Now when I am surrounded by enemies worse than starving footpads and assassins, when the Dutchman Orange would, they say, go down on his knees and thank God for my taking off, when the ministers of my imbecile brother-in-law Charles of Spain would have me assassinated on my own hearth if it could be accomplished; when there is not a country in all Europe, except that over which Charles Stuart now reigns, that does not thirst for my life. Indeed I need good friends like you, Louis, and you, Ludovois. In the one to whom I have confided the charge of my own guards, the other the care of my own army." "Your Majesty may rely on me and my guards," de Beaurepaire said. "Your Majesty may rely on——" "I know, I know," Louis said, "Should I have confided that charge to you otherwise?"

De Beaurepaire learns later that, by the heroism of a young Englishman who, after being stabbed and thrown into the Rhine at Basle by the conspirators when they learn he has overheard their plans, had yet, though apparently at death's door, made his way to Fontainebleau and into the King's presence, revealing the whole of the conspiracy and the names of those engaged in it. The true nature of the man who had permitted others to name him as leader rather than placed himself in that position, is shown in the following passage when he has allowed the wounded Englishman to pass him unmolested, knowing at the same time that he was in possession of information that would spell ruin to him: "It could be done in a moment. None would ever know . . . that was all that was needed! Yet was it all? . . . Ah! God! Would He not know always? Would his own heart not know? Yes, always! Always! He would become a two-fold murderer. And he was—a de Beaurepaire! With a sound that . . . might have been a curse—or a sob—he loosed his rein—and rode away to Paris. Away to Paris to meet his confederates . . . to tell them that they were betrayed . . . while he, their chief, . . . had had the chance of slaying him—and had let that chance pass." "Traitor and True" is an admirable specimen of the well-abused but always popular historical novel.

* "Traitor and True." By J. Bloundelle Burton. (John Long. 6s.)

### Notes and Queries.

**REGULATIONS.**

**The Editor is always willing to answer in his olumn, without any fee, all reasonable questions, as soon as possible.**

**But the following rules must be carefully observed.**

**1. Every communication must be accompanied by the name and address of the writer.**

**2. The question must always bear upon nursing, directly or indirectly.**

**If an answer is required by letter a fee of half-a-crown must be enclosed with the note containing the inquiry.**

*Maternity Nurse.*

(73) I am married. Can I train at a maternity hospital? Where can I train? What fees? Can I practise without becoming a midwife? What books should I study?—*Chiswick.*

Married women are accepted. The Maternity Nursing Association, 5 Little James Street, Bedford Row, W.C., trains for £10 to £25, and gives certificates for monthly nursing. You need not be a midwife. We advise you to obtain "A Complete Handbook of Midwifery," by J. K. Watson, post free 6s. 4d., and "How to Become a Certified Midwife," by E. L. C. Appel, post free 1s. 1d., Scientific Press, 28 & 29 Southampton Street, Strand, W.C.

*Electrolysis.*

(74) Is electrolysis a permanent remover of the hair, and does it leave a scar?—*M. E. G.*

Electrolysis is the most permanent method, and if properly done leaves no scar.

*Age for Training.*

(75) I am not nineteen yet. I should like to know if I can get into a hospital at once, or how soon.—*King's Cross.*

You can be received at some special hospitals at twenty. You had better get "How to Become a Nurse," 2s. 4d. post free, from the Scientific Press, 28 Southampton Street, Strand.

*State Registration.*

(76) Can you tell me if State registration will be established in January?—*Busy.*

No Bill is passed yet, and no one can predict if or when one will be passed.

*Helpless.*

(77) Is there any bed suitable to a quite helpless person which precludes the necessity of rising from bed when alone?—*Matron.*

Yes. Write to any of the invalid furniture firms advertised in our columns, and they will help you.

*Number of Beds.*

(78) Can you tell me the number of beds in the eight hospitals the names of which I enclose?—*Audacious.*

You will find all the information you require in Burdett's "Hospitals and Charities," published by the Scientific Press, 28 Southampton Street, Strand, W.C. Price 6s.

*Lip Reading.*

(79) Can you tell me of anyone teaching lip reading to the deaf.

Write to one of the deaf and dumb institutions. They will be able to help you.

*India.*

(80) Can you tell me how to apply for an appointment to Lady Roberts' Staff in India.—*Lewisham.*

There are no vacancies in Lady Roberts' Nursing Service, full particulars of which appeared in our issue of October 13, 1906; but you can obtain any further information you require from the Lady Superintendent, The Hospital, Murree, India.

*Central Africa.*

(81) Where should I apply for an appointment in the Shiri Highlands Railway Hospital, Chiromo?—*Central Africa.*

Write direct to the hospital.

**Handbooks for Nurses.**

| | Post Free. |
|---|---|
| "How to Become a Nurse: How and Where to Train." | 2s. 4d. |
| "Nursing: its Theory and Practice." (Lewis.) ... | 3s. 6d. |
| "Nurses' Pronouncing Dictionary of Medical Terms." | 2s. 6d. |
| "Complete Handbook of Midwifery." (Watson.) ... | 6s. 4d. |
| "Preparation for Operation in Private Houses." ... | 0s. 6d. |

Of all booksellers or of The Scientific Press, Limited, 28 & 29 Southampton Street, Strand, London, W.C.

### For Reading to the Sick.

### AT REST.

When the weary ones we love
Enter on their rest above,
Seems the earth so poor and vast,
All our life-joy overcast?
Hush, be every murmur dumb:
It is only till He come.

*Bishop Bickersteth.*

Those whom the world calls dead are sleeping, because they are taking their rest. "I heard a voice from heaven saying unto me, write, Blessed are the dead which die in the Lord, from henceforth. Even so saith the Spirit; for they rest from their labours." Not as the heretics of old vainly and coldly dreamed, as if they slept without stir of consciousness from the hour of death to the morning of the resurrection. Their rest is not the rest of stone, cold and lifeless; but of wearied humanity. They rest from their labours; they have no more persecution, nor stoning, nor scourging; no more martyrdoms; they have no more false witness, nor cutting tongues; no more bitterness.

Blessed and happy dead! In them the work of the new creation is well nigh accomplished. What feebly stirs in us, in them is well-nigh full. They have passed within the veil, and there remaineth only one more change for them—a change full of a foreseen, foretasted bliss. How calm, how pure, how sainted, are they now! A few short years ago, and they were almost as weak and poor as we: harassed by temptations, often overcome weeping in bitterness of soul, struggling, with faithful though fearful hearts, towards that dark shadow from which they shrank as we shrink now.

Again, death is changed to sleep, because they whom men call dead do really live unto God. They were dead while they lived this dying life on earth, and dead when they were in the last avenues of death. But after they had once died, death had no more dominion: they escaped as a "bird out of the snare of the fowler"; the snare "was" broken, and they were delivered.—*H. E. M.*

The blessed dead in Paradise are at rest, to sum up much in the fewest words. This is a distinct revelation of Holy Scripture, and one which we might have been sure of, even without its direct testimony. In life they took their Master at His word; "wearied and heavy-laden"—as all at times must consciously be—"they came to Him, and He gave them rest." Their being at rest is only a further, higher, stage of this; not sleep, a passing away, a time of waiting in objectless tranquillity. There is evidence that former faculties may have received marvellous accessions of power. Although their state is not one of perfect fruition. . . . They know that they are growing more fit for the full fruition of God's Presence, more meet for the inheritance of the Saints in light.—*R. G. Swayne.*

Hold them safely, free from torment,
In that waiting place,
And those loving Hands direct us
On our earthly race;
One communion—theirs, the haven,
Ours, the narrow road;
They are kept, and we are guided
By the Hands of God.

*A. R. Greenaway.*

